# The Anti-Inflammatory and Antibacterial Action of Nanocrystalline Silver and Manuka Honey on the Molecular Alternation of Diabetic Foot Ulcer: A Comprehensive Literature Review

**DOI:** 10.1155/2015/218283

**Published:** 2015-07-28

**Authors:** Ka-Kit Tsang, Enid Wai-Yung Kwong, Kevin Y. Woo, Tony Shing-Shun To, Joanne Wai-Yee Chung, Thomas Kwok-Shing Wong

**Affiliations:** ^1^O&T Department, Queen Elizabeth Hospital, Hong Kong; ^2^Department of Nursing, The Hong Kong Polytechnic University, Hong Kong; ^3^School of Nursing, Faculty of Health Sciences, Queen's University, Kingston, ON, Canada; ^4^Department of Health Technology & Informatics, The Hong Kong Polytechnic University, Hong Kong; ^5^The Faculty of Liberal Arts and Social Sciences, The Hong Kong Institute of Education, Hong Kong; ^6^Ginger Knowledge Transfer and Consultancy Limited, Hong Kong

## Abstract

Honey and silver have been used since ancient times for treating wounds. Their widespread clinical application has attracted attention in light of the increasing prevalence of antibiotic-resistant bacteria. While there have been a number of studies exploring the anti-inflammatory and antibacterial effects of manuka honey and nanocrystalline silver, their advantages and limitations with regard to the treatment of chronic wounds remain a subject of debate. The aim of this paper is to examine the evidence on the use of nanocrystalline silver and manuka honey for treating diabetic foot ulcers through a critical and comprehensive review of in vitro studies, animal studies, and in vivo studies. The findings from the in vitro and animal studies suggest that both agents have effective antibacterial actions. Their anti-inflammatory action and related impact on wound healing are unclear. Besides, there is no evidence to suggest that any topical agent is more effective for use in treating diabetic foot ulcer. Overall, high-quality, clinical human studies supported by findings from the molecular science on the use of manuka honey or nanocrystalline silver are lacking. There is a need for rigorously designed human clinical studies on the subject to fill this knowledge gap and guide clinical practice.

## 1. Introduction

The use of silver as a prophylactic and treatment for infections and other diseases dates back to about 100 BC, when it was used for this purpose by the ancient Greeks and Romans [1]. In the late nineteenth century, there was a resurgence of interest in using silver compounds to treat venereal diseases and eye infections [2]. The topical application of honey was also a common practice for centuries [3] by the Egyptians, Greeks, Romans, and Chinese [4]. The early Egyptians of around 1650 BC were the first to use honey as a component in the topical treatment of wounds, as evidenced from the text of the Smith papyrus [5]. A document from 1392 details wound care practices, including the use of honey, in the Middle Ages [6]. However, the use of honey came to be considered outmoded around the 1940s, following the advent of antibiotics. With the recent increase in multiresistant bacteria due to the overuse of antibiotics in the past few decades, the potential of honey and silver in the management of various chronic wounds such as diabetic foot ulcers, venous ulcers, and pressure ulcers has spurred new interest in the wound care community [7].

## 2. The Challenge of Managing Diabetic Foot Ulcers

Diabetic foot ulcer (DFU) is the focus of the present review because of the high prevalence of the disease and the burden it places on the health system. It is estimated that diabetes affects 8.3% of the global population or 382 million people [8]. This number continues to grow, making DFU a major public health problem [9]. Foot ulcer is a common complication, affecting 4%–10% persons with diabetes mellitus [10] and preceding over 85% of amputations in this population of patients [11]. The risk of complication increases with time. The cumulative incidence of DFU increases from 27.3% during the first year of diagnosis to 76.4% five years after the initial diagnosis. The rate of amputation increases from 12.5% to 47.1% [12].

The cost of caring for people with diabetes is exorbitantly high, amounting to $174 billion in the United States in 2007, of which foot ulceration accounted for 24% to 31% [13]. Stockl et al. [14] revealed that the average cost per DFU episode was $13,179, and greater in the case of deep ulcers with coexisting infection and circulation problems (as evaluated using the Wagner classification system). Apart from the financial impact of DFU, patients with DFU experience many limitations in their physical, social, and vocational activities (especially those who were required to undergo an amputation), leading to poor health-related quality of life (HRQOL) [15, 16].

There are multiple factors behind the development of DFU, with neuropathy, peripheral vascular disease (PVD), and foot deformity being the most prominent risk factors [17, 18]. The lack of protective sensation predisposes a person with diabetes to suffering from repetitive trauma and ulcers that do not heal due to poor circulation [19]. Wound healing can be further complicated by bacterial infections [20, 21] and prolonged inflammation [22–25]. The reasons for such prolonged and excessive inflammation are still unclear [22, 26], but they likely involve bacterial colonization, biofilms, and recurrent tissue trauma [27]. Apart from the obvious clinical predisposing risk factors, recent studies have revealed that complex cellular and molecular aberrations such as poor extracellular matrix (ECM) formation, high levels of matrix metalloproteinases (MMPs), and other proinflammatory cytokines, as well as oxidative stress, are responsible for delayed healing [28].

### 2.1. Cellular Abnormalities

The exact mechanisms behind poor wound healing remain elusive [29]. Loots et al. [30] showed a diminished proliferative capacity and an abnormal morphology of fibroblasts in wounds related to diabetes. Galkowska et al. [31] found in an in vitro study that the healing process of diabetic foot ulcers may be hampered by mechanisms that reduce the accumulation of leukocytes. Waltenberger et al. [32] performed a chemotaxis assay using isolated monocytes from diabetic patients and found that monocytes are less responsible for the vascular endothelial growth factor (VEGF) when compared with normal person. Using immunohistochemistry techniques, Usui et al. [33] discovered that keratinocyte migration and differentiation were impaired along the margin of chronic ulcers in patients with diabetes mellitus. Albiero et al. [34] discovered that wound healing delayed as a result of diabetes was associated with the defective recruitment, survival, and proliferation of BM-derived endothelial progenitor cells in mice. Macrophages isolated from diabetic mice also exhibit greater infiltration by inflammatory M1 macrophages and may contribute to impaired diabetic wound healing [35].

### 2.2. Poor ECM Formation and High Levels of MMPs

The ECM formation is defective in DFU. ECM creates a scaffold for cellular attachment, which is crucial for wound healing. Blakytny and Jude [29] stated that the disruption in the formation of new ECM as well as the diminished stimulation of cell proliferation results in the lack of a proper scaffold for cellular attachment.

MMPs are zinc-dependent endopeptidases, and their inhibitors are called tissue inhibitors of matrix metalloproteinases (TIMPs). They are excreted by a variety of connected tissue, fibroblasts, keratinocytes, proinflammatory cells such as neutrophil, and macrophage. Those MMPs are regulated by hormones, growth factors, and cytokines in response to signals [36, 37]. The functions of MMPs include influencing cell migration, promoting cellular proliferation apoptosis, modulating growth factors and their receptors, and degrading the structural components of ECM during the remodeling of tissue [37, 38].

In diabetic patients, hyperglycaemia activates the pathways of the mitogen-activated protein kinase to stimulate the production of cytokine and promote inflammation [39]. The high level of MMPs is also the pathological alternation in DFU in biochemical terms. The overexpression of MMPs and elastase breaks down the components of ECM and inhibits growth factors [40]. Lobmann et al. [41] compared the MMP levels of 20 patients with diabetic foot ulcers with those of 12 patients with traumatic ulcers. The results showed that the concentrations of MMP-1 and MMP-9 increased 65-fold and 14-fold, respectively, in the diabetic ulcer biopsies. Muller et al. [42] conducted another cohort study on sixteen patients with neuropathic diabetic ulcers. The results echoed those of Lobmann's study. The levels of MMP-8 and MMP-9 decreased in the good healer group and remained stable in the poor healer group during the 12-week follow-up period.

### 2.3. High Proinflammatory Cytokines

To heal ulcers, proinflammatory cytokines can chemotactically draw inflammatory cells into the injured area [43]. Lobmann et al. [27] stated that the upregulation of TNF-*α* and IL-1 stimulates the synthesis of MMP-1 and inhibits the synthesis of collagen. Trengove et al. [44] found in an in vitro study the upregulation of interleukin-1 (IL-1), interleukin-6 (IL-6), and tumor necrosis factor-alpha (TNF-*α*) in chronic nonhealing ulcers. On the other hand, decreased levels of all proinflammatory cytokines can improve wound healing. Chan et al. [45] found in an in vitro study that the neutralization of TNF in diabetic wounds improves the angiogenesis.

### 2.4. High Oxidative Stress

People with diabetes usually have hyperglycemia. There is increasing evidence to suggest a causal link between hyperglycemia and oxidative stress leading to cellular damage [40]. Excessively high levels of free radicals cause damage to cellular proteins, membrane lipids, and cell nucleic acids and eventually lead to cell death [46]. In people with diabetes, free radicals (superoxide anion and hydroxyl radial) are formed in disproportionately high levels by glucose oxidation, the nonenzymatic glycation of proteins, and the subsequent oxidative degradation of glycated proteins. The glycated proteins develop further reactions to form advanced glycation end products (AGEs) [47, 48]. The accumulation of AGEs causes the upregulation of proinflammatory cytokines and MMPs that will degrade ECM through the production of reactive oxygen species (ROS) [29]. The production of peroxynitrite anion and peroxynitrous acid [49] can also lead to biological damage [50]. The molecular alternations of DFU are summarized in Table 1.

Because of the molecular alternations of DFU shown in Table 1, DFU is difficult to treat clinically. Many clinicians attempt to apply different topical dressing materials to treat DFU. However, few high-quality studies have been conducted to guide clinical practice on the application of topical dressing materials. In the recent Cochrane database on systematic reviews, no papers related to honey and silver met the inclusion criteria in this review because of the poor quality of the papers [51, 52]. Even though many randomized controlled trials have recently been conducted, they suffer from methodological flaws related to the design of the research, including a small sample size, deficient randomization or blinding, and poor statistical analysis. Moreover, it is difficult to pool together and analyze trials on dressing materials because of the diversity of the studies [53]. At the same time, honey and silver are widely used by clinicians to treat DFU because of their antibacterial effect. In the present review, we examine the evidence relating to the anti-inflammatory and antibacterial actions of nanocrystalline silver (nAg) and manuka honey (MH) to determine their effects on the molecular alternation of DFU.

## 3. Action of Nanocrystalline Silver

### 3.1. Antibacterial Effect of nAg

nAg can increase the surface area that is in contact with the wound surface [54]. The bactericidal effect of nAg depends on its size. It is preferable for nAg to be around 1–10 nm in size, as this is the size at which direct interaction can occur with the surface of the cells and within the bacteria [55]. Thus, nAg can increase the bioactivity and solubility of silver to allow chemical reactions to take place [112]. Apart from size, the shape of nAg can also affect their bactericidal effect. Pal et al. [56] found that the bactericidal action of a truncated triangular shape exceeds that of a spherical as well as a rod shape. As regards antibacterial action, Lok et al. [113] revealed the exposure of* Escherichia coli* (*E. coli*) to nAg for a short time. They discovered that the mechanisms of nAg and silver ions were similar but that nAg appears to be significantly more efficient than silver ions. nAg and Ag^+^ share the following common bactericidal effects. Silver ions can interact with thiol group-containing enzymes, such as NADH-dehydrogenase II in the respiratory system [57]. This will lead to the formation of hydroxyl radicals and to an attack on the cell itself and subsequently to DNA damage [58]. Besides, silver ions induced apoptotic pathways inside the bacteria, leading to their death [54, 56]. Pandian et al. [59] conducted an in vitro study to echo the apoptotic pathways. They found that bacteria that showed evidence of apoptosis and deoxyribonucleic acid (DNA) were fragmented after exposure to nAg.

In vitro evidence shows that nAg^+^ has a unique antibacterial action on cells. There is an electrostatic attraction between nAg^+^ and the negative charge cell membranes of bacteria. nAg binds to the modified phospholipid bilayer and induces a massive leakage of protons [60]. When nAg^+^ anchors to the bacterial cell wall and causes structural change by forming irregular-shaped “pits” on the bacterial outer membrane, the permeability of the membrane changes and becomes porous [61]. In an electron spin resonance spectroscopy study, Kim et al. [62] further discovered that that nAg generates free radicals, which can damage the bacterial cell membrane. The cell will then progressively release lipopolysaccharides and membrane protein, which will ultimately cause the cell to die. Mirzajani et al. [63] further investigated the effect of nAg on Gram-positive* Staphylococcus aureus*. They discovered that nAg exerted on the bacterial peptidoglycan cell wall and specifically decomposed its peptide part. By attaching to the bonds of glycan strands composed of N-acetylglucosamine and N-acetylmuramic acid, nAg destroyed the bonds and released muramic acid. As a consequence, “pits” were generated on the bacterial cell membrane. However, the exact mechanism that occurred on the Gram-negative membrane remained unknown. McQuillian et al. [64] continued working on Gram-negative bacteria and investigated the effect of nAg on* Escherichia coli*. Using inductively coupled plasma mass spectrometry, they discovered that the primary mechanism of nAg was to interact with and dissolve the outer and inner membranes of a cell; then Ag^+^ released into the cell and affected a transcriptional response.

In addition, nAg can dephosphorylate the peptide substrate on tyrosine residues. Mijakovic et al. [65] demonstrated that the phosphorylation of protein substrate in bacteria can influence bacterial sign transduction. Shrivastava et al. [66] found that the inhibition of this mechanism by nAg can influence the signal transduction and stop the growth of bacteria. In summary, the overall effect of nAg is both to inhibit the reproduction of bacteria and to kill them directly.

### 3.2. Bacterial Resistance to Silver

However, some reports have documented bacterial resistance to silver on possible wound pathogens. The pathogens included* Providencia stuartii* [114],* Enterobacter cloacae* [115],* Pseudomonas* species [116],* Acinetobacter baumannii* [117], and* E. coli* [118]. Laboratory studies found that the efflux of silver ions or the acquisition of genetic material in the form of plasmids was the mechanism of a silver-resistant* E. coli* mutant [119].

Some researchers have claimed that the clinical incidence and probability of multiple mutations remain low even though silver resistance has been documented [120, 121]. Unlike antibiotics, silver attacks more than one of the target specific sites [122] and there is no direct evidence that silver resistance mechanisms confer cross-resistance to antibiotics [120]. Percival et al. [123] continued the work on Ag-containing wound dressings in vitro. They concluded that despite evidence of genetic resistance to silver, all strains were killed following a maximum of 48 hours of exposure to the dressings.

### 3.3. Anti-Inflammatory Effect of nAg

Compared with the antibacterial action of silver, the exact mechanism of its anti-inflammatory action is still unclear. To our knowledge, the studies that have been published on the subject have focused on the observable effect of the anti-inflammatory action of silver, instead of on the detailed molecular mechanism. The animal and in vivo evidence showing the anti-inflammatory effect of nAg are as follows. Wright et al. [67] discovered that a full-thickness contaminated wound in a porcine model treated with nAg healed more quickly and was accompanied with a decrease in MMP activities as well as with the stimulated apoptosis of polymorphonuclear leukocytes (PMNs). They suggested that enhanced cellular programmed cell death (apoptosis) would decrease the number of cells to release cellular content with numerous cytotoxic compounds such as proteases, oxygen radicals, and various acids, in turn decreasing local inflammation. Bhol et al. [68] had similar findings. Through clinical observations, they found that topical nAg cream was effective at decreasing allergic contact dermatitis on a guinea pig model and that the effect was similar to that achieved using topical steroids and immunosuppressive drugs. Bhol and Schechter [69] further extended their work using a rat model. From a histological and immunohistochemical examination, they discovered that nAg had the ability to inhibit contact dermatitis by suppressing TNF-*α* and IL-12, as well as inducing the apoptosis of inflammatory cells. Similar to previous findings, Wong et al. [70] showed that nAg was effective at decreasing inflammation through the downregulation of the production of TNF-*α*, without a significant toxic effect on a peritoneal adhesion model of mice.

Nadworny et al. continued the work that had been conducted on the effect of nAg by using different animal models. The results echoed the previous findings. They further discovered that nAg had the ability to promote healing because some kinds of growth factors were elevated after the topical nAg intervention. Using a porcine model, Nadworny et al. [71] found that treatment with solutions containing nAg resulted in a decrease in proinflammatory cytokines TNF-*α* and IL-8 expression, with an increase in IL-4, epidermal growth factor (EGF), keratinocyte growth factor (KGF), and KGF-2 expression. Again using a porcine model, Nadworny and colleagues [72] also discovered that nAg was mainly deposited in the epidermis but that cell apoptosis was large in the dermis and minimal in the epidermis. They therefore proposed that the anti-inflammatory effects of nAg were induced by interactions with cells in the top layers of the skin, which then released biological signals resulting in widespread anti-inflammatory activity. nAg induced the downregulation of TNF-*α* and IL-8 expression, as well as the upregulation of IL-4, IL-10, EGF, KGF, and KGF-2. Bisson et al. [73] furthered the work on the latest nAg topical dressing for a histopathological analysis involving mice. The result demonstrated that nAg dressings have a significant inflammatory effect on a noninfected inflammatory skin model, equivalent to that obtained using topical steroid cream.

In addition, there is some in vivo evidence showing that nAg has an anti-inflammatory effect. Shin et al. [74] performed the work in human cells. The result showed that TNF-*α* and interferon-*γ* were significantly inhibited at low concentrations of nAg of over 3 ppm. Mani et al. [75] examined the effect of synthetic nAg on healthy human cells. They also showed that all three cytokines (TNF-*α*, IL-1*β*, and IL-6) were inhibited at concentrations ranging from 10 to 20 *μ*g/mL. Obviously, from the published animal and human studies, the anti-inflammatory effect of nAg is clear although the exact pathway is still unknown. Interestingly, all histological findings demonstrated apoptosis of the inflammatory cells induced by nAg, so that avoiding the inflammatory cells produces chemoattractants to induce further inflammation upon bursting. In addition, all of the molecular findings indicated that the TNF-*α* expression was downregulated. In diabetic wound healing, impairing the proliferation of fibroblasts has been linked to an increase in the level of TNF-*α* [124]. When the level of TNF-*α* level was inhibited, there was increase in the number of fibroblasts proliferation significantly [125]. Therefore, we can hypothesize that the anti-inflammatory effect of nAg may be beneficial to the healing of DFU. The actions of nAg are summarized in Table 2.

### 3.4. Clinical Evidence on the Use of nAg-Impregnated Dressings on DFU

To date, no unifying theories have been established through the above basic science research and brought into the clinical context. Nevertheless, researchers have investigated Ag or nAg topical dressings and tried to link their findings with the observable clinical outcome. Unfortunately, there have been few clinical studies on the effect of nAg on DFU. We searched different databases for studies on the effect of Ag or nAg dressings on DFU that had been published in the past 10 years and identified one case report [126], two case series studies [127, 128], two randomized controlled trials (RCT) [129, 130], and one meta-analysis [52]. Since the effect of nAg is more potent than Ag and the molecular mechanism is not exactly the same, the non-nAg studies were excluded from the analysis. Only one case series study investigated the effect of nAg. Cahn and Kleinman [127] used a nonsurgical approach to treat six patients with diabetic foot abscesses. The abscesses were treated with topical oxygen and drained using nAg foam rope (Polymen Wic Silver Rope). The effect on debridement and oxygen therapy on the diabetic foot abscesses could not be excluded. In this case series study, the patients varied in terms of their history of diabetes (5 to 20 years) and ankle brachial index score (0.57 to 1.63). The duration of their treatment ranged from two to eight months. In addition, three patients suffered from peripheral vascular disease. During the period that they were being treated for diabetic foot abscesses, they also underwent percutaneous revascularization. Although all of the patients with diabetic foot abscess and osteomyelitis had completely healed within two to nine months by treatments using debridement, a topical oxygen extremity chamber, and Polymen Wic Silver Rope, we could not determine the sole effect of nAg foam in this study.

### 3.5. Cytotoxicity Effect of Silver on Modern Wound Dressings

The cytotoxicity of silver is an issue that has been debated. Lansdown [131] claimed that toxicity from dressings containing silver is rare because in modern dressings the silver is in a controlled-release preparation and some of the silver ions bind to the protein of the wound exudate. On the other hand, in vitro and animal studies have shown that silver dressings have significant cytotoxic effects on keratinocytes and fibroblasts [132, 133]. Van den Plas et al. [134] found that silver dressings induced rapid cell death within two hours; they recommended the use of silver dressings only on critically contaminated wounds. Zou et al. [135] compared different pairs of Ag-based and non-Ag-based dressings with basic materials in vitro. The result showed that human fibroblasts, which were extracted from diabetic patients, decreased in viability by 54–70% and collagen synthesis by 48–68% when they came into contact with the Ag-based dressings compared with the non-Ag-based dressings. They did not suggest discarding Ag dressings but stated that such dressings should be used with caution when treating noninfected diabetic wounds. Therefore, the international consensus on the use of silver is that silver should be discontinued if wound infection is no longer present [136].

However, the findings from recent human studies do not support the view that using modern silver dressings could lead to cytotoxicity. Karlsmark et al. [137] noted that the serum silver level for patients treated with a silver dressings was no higher than the reference value, although five patients experienced a temporary increase in their silver level. Gago et al. [138] provided further evidence that a high level of Ag rapidly reduces infection and results in the faster healing of infected chronic wounds. Lansdown et al. [139] discovered that the ions from a silver dressing penetrate only several millimeters into the wound bed. The available information indicates that the findings from the in vitro studies are inconsistent and that knowledge on the cytotoxicity of Ag and nAg dressings is incomplete. Based on our existing knowledge, it can be said that silver has both anti-inflammatory and antibacterial properties. Indeed, toxicity from the long-term use of silver in clinical practice cannot be completely ruled out, especially when silver is used as an anti-inflammatory moderator instead of an antibacterial agent. Further research is needed in this area to ensure the clinical safety of using silver dressing therapy over the long term [140]. There is both a knowledge gap and a clinical query with regard to the use of silver as an anti-inflammatory moderator for treating wounds with no infection but with inflammation.

## 4. Action of Manuka Honey

### 4.1. Antibacterial Action of Manuka Honey

MH, which comes from* Leptospermum scoparium* in New Zealand, exhibits antibacterial activity [76]. Our body of knowledge on its antibacterial mechanism remains incomplete. The antibacterial action of MH is mainly based on its physical properties and on the active ingredient that it contains. First, honey has an osmotic effect, drawing moisture from the environment and dehydrating bacteria [76]. This effect is reduced after dilution by wound exudate [141]. Second, the pH value of MH is between 3.2 and 4.5. This acidic nature can inhibit the growth of most microorganisms, such as* E. coli*,* Pseudomonas aeruginosa* (*P. aeruginosa*),* Salmonella* species, and* Streptococcus pyogenes* [77, 142, 143]. Third, methylglyoxal (MGO) is one of the phytochemical factors with antibacterial activity that have been identified within MH. In vitro studies have revealed that the MGO in MH contains the majority of the nonperoxide activity and that the amount of MGO is closely related to the level of antibacterial activity [78, 79]. Ordinary honey has a limited amount of MGO, ranging in concentration from 1.6 to 135 mg/kg, compared to 38–725 mg/kg in manuka honey [78]. A minimum inhibitory concentration (MIC) for* E. coli* and* Staphylococcus aureus* (*S. aureus*) was observed at 1.1 mM of MGO [79]. Atrott and Henle [80] also found a linear correlation between MGO levels in 61 samples of manuka honey and antibacterial activities. There were also some other unidentified biochemical substances in the MH, which later laboratory studies revealed to also contribute to antimicrobial activity. Kwakman et al. [81] identified some cationic and noncationic compounds that contributed to bactericidal activities against different types of bacteria after the neutralization of MGO. Kato et al. [82] found a glycoside of methyl syringate called “Leptosin,” which had a positive correlation with antibacterial activity in MH.

Unfortunately, the exact mechanism contributing to the bactericidal activity of MH remains largely unknown [78]. The antibacterial action of MH was recently explored with the hope of elucidating the related mechanism, but its precise mode of antibacterial action is only just beginning to be understood. In vitro studies have demonstrated that cell division is interrupted and cell separation cannot occur following the formation of septa on* S. aureus* [83] and MRSA [84] when these bacteria are exposed to MH. Roberts et al. [85] observed extensive cellular lysis of* P. aeruginosa* at an MIC concentration of 12% w/v in MH. The honey-treated cells were unable to form microcolonies and two target genes were identified as being involved in the process. In addition, Packer et al. [86] found that MH causes two different proteins to be downregulated and one to be upregulated on* S. aureus*. These two proteins had roles to play in ribosomal function, protein synthesis, the metabolic process, and transcription, indicating that MH could interfere with the ribosome or its translational capacity. Merckoll et al. [144] discovered that honey has bactericidal effects on both planktonic and biofilm-embedded bacteria, since bactericidal substances in honey can penetrate into the biofilm. Maddocks et al. [87] further found that MH decreases the formation of biofilm by inhibiting the* Streptococcus pyogenes* from binding to fibronectin. It has this effect because this binding is important for the colonization of bacteria and the development of biofilm [145]. Iron is necessary to sustain the growth of bacteria.* P. aeruginosa* produced two extensively characterized siderophores to capture iron [146]. Kronda et al. [88] further discovered that MH decreased the production of siderophores at both 1/4 and 1/2 MIC, showing that MH impeded the growth of the cells. So far, the above in vitro evidence suggests that there is no single mechanism to antimicrobial action but that a combination of factors results in diverse modes of antibacterial inhibition and killing.

Compared with antiseptics that decrease the bacterial count within minutes, the antibacterial activity of MH is much slower [146]. The latest laboratory studies explain this phenomenon. Kwakman et al. [81] showed that MH does not have a rapid antibacterial effect against different kinds of bacteria in the first 2 hours but that its potency increases after 24 hours. The reason for the slow onset action relates to the fact that MH lacks major factors involved in rapid antibacterial activity, such as bee defensin-1 and hydrogen peroxide. The main active ingredient in the antibacterial effect of MH is MGO. Adams et al. [147] found that MGO forms through the conversion of dihydroxyacetone. This conversion is a nonenzymatic reaction that takes place in the presence of proteins or amino acids. The concentration of MGO is low in freshly produced honey but increases after storage at 37°C.

MH has an anti-antibacterial effect on different microorganisms in vitro. The rate of inhibition depends on the species of bacteria and the concentration of honey [141, 148]. Cooper et al. [89] found that MH would still prevent the growth of* S. aureus* if diluted by a further 7- to 14-fold in vitro. Cooper et al. [90] continued the work and discovered that 17 strains of* pseudomonas* isolated from infected burn wounds could be killed by MH, even when diluted more than 10-fold. Hammond and Donkor [91] investigated the bactericidal effect of* Clostridium difficile* on MH. They found that the corresponding MIC and minimal bactericidal concentration (MBC) were both 6.25% (v/v). Kwakman et al. [81] further discovered that the bactericidal activity of MH could kill* Bacillus subtilis*,* P. aeruginosa*, and* E. coli*. Maddocks et al. [87] identified the bactericidal effect of MH on* Streptococcus pyogenes* in both planktonic cultures and biofilm. Apart from that, MH can also kill antibiotic-resistant bacteria. Cooper et al. [92] found that seven strains of vancomycin-resistance* Enterococci* were inhibited by MH at 4.61 ± 0.51% (v/v). French et al. [93] demonstrated that MH inhibited 18 strains of antibiotic-resistant coagulase-negative* Staphylococci* at dilutions of down to 29.9 ± 1.9% (v/v). Interestingly, MH has a synergistic antibacterial effect with antibiotics. Five antibiotics and MH combinations were found that improve antibacterial effectiveness in vitro [149]. However, MH cannot kill all microorganisms. Lusby et al. [150] revealed that MH is unable to inhibit* Serratia marcescens* and* Candida albicans*.

However, the effect of MGO in diabetic ulcers is debatable. Price and Knight [151] pointed out that MGO changed the structure and function of immunological enzymes to form AGEs and reduced the efficiency of the peripheral blood immune-cell response. Majtan [152] further pointed out that manuka honey contains high levels of MGO and speculated that patients with diabetes may be placed at risk by the use of manuka honey because of its direct negative effect on cells or its indirect effect on the formation of AGEs, which could impair the wound-healing process. In an in vitro study, Sassi-Gaha et al. [153] found that highly reactive dicarbonyls attacked the lysine, arginine (Arg), and cysteine residues of long-lived proteins (e.g., collagens) to form irreversible AGEs, causing changes in collagen pathophysiology. Therefore, there is clearly a paucity of high-quality human studies relating to the use of topical honey to treat diabetic ulcers.

### 4.2. Anti-Inflammatory Action of Manuka Honey

Honey has been shown to reduce both acute and chronic inflammation, although the mechanism for this anti-inflammatory action is not entirely understood [154]. The antioxidants found in honey are considered to be important determinants of anti-inflammatory activity [155]. The antioxidant properties of honey are beneficial in counteracting advanced glycation and lipoxidation end products, which can induce oxidative stress and inflammation in diabetics [156]. Natural honey contains flavonoids, phenolic acids, and other enzymes. All of the active components work together to provide a synergistic anti-inflammatory and antioxidant effect [157]. Chan et al. [94] revealed that pinobanksin, pinocembrin, luteolin, and chrysin were the major flavonoids found in MH, accounting for 61% of the total flavonoids. Low levels of quercetin and galangin were also found. Flavonoids are known for their anti-inflammatory activity. Cho et al. [96] found that chrysin was able to suppress the activity of proinflammatory enzymes, cyclooxygenase-2 (COX-2), and inducible nitric oxide synthase (iNOs). Raso et al. [95] discovered that several flavonoids, including quercetin and galangin, also inhibited the expression of COX-2 and iNOs in a concentration-dependent manner.

Honeys from different botanical origins have different components. The following in vitro studies give a clear picture of the anti-inflammatory action of MH. Henriques and colleagues [99] discovered that MH had the strongest antioxidant capacity among the varieties of honey that were tested and that it was able to quench the added free hydroxyl radicals within 5 minutes of being added. This antioxidant capacity contributed to the ability of MH to resolve chronic inflammations, including ulcers. In addition, Tonks et al. [100] revealed that MH stimulated both the proinflammatory cytokines TNF-*α* and IL-1*β* and the anti-inflammatory cytokine IL-6 from monocytes. Tonks and his colleagues [97] further found that a 5.8 kDa component isolated from MH stimulated these cytokines via toll-like receptor (TLR) 4. After heat treatment sugar syrup or MH loses this function. This explains why supermarket honey cannot be used as medicinal honey for treating wounds. van den Berg et al. [47] also discovered that the phenolic constituents of MH were able to inhibit the production of ROS and scavenge superoxide anion. Recently, researchers from the University of Waikato investigated the anti-inflammatory activity of MH in vitro. Bean [101] found that MH increased both the expression of the proinflammatory cytokine TNF-*α* and the anti-inflammatory cytokines Il-10 and IL-1 and the growth factors PDGF and TGF-*β*. ROS production by phagocytosis was also downregulated in the presence of MH. These findings are in alignment with previous in vitro studies and explain why MH may allow inflammation to proceed at a modulated level while simultaneously allowing healing to occur.

Leong et al. [102] determined that MH decreased the production of superoxides by neutrophil in vitro and decreased odema and leukocyte infiltration in a mice model but were unable to pinpoint the specific content contributing to the inflammatory action. Tomblin et al. [98] further discovered that this anti-inflammatory activity of MH directly correlated to the phenolic content through a TLR1/TLR2 signalling pathway. The higher phenolic content produced an elevated anti-inflammatory effect. This result echoed the findings of Leong et al. [102] on the specific content inside MH that is responsible for the anti-inflammatory function. The antibacterial and anti-inflammatory actions of MH are summarized in Table 3.

### 4.3. The Clinical Evidence on the Use of MH on DFU

In DFU, the local factors that hinder wound healing are a high bioburden and a high inflammatory response. MH has been confirmed to have such properties in vitro. Unfortunately, there is limited high-quality evidence to show its effect in vivo. We reviewed single case studies, case series, and randomized controlled trials on the effect of honey or related products that had been published in the past 10 years. Six single case studies [158–163], five case series studies [164–168], one controlled trial [169], and three RCTs [104, 105, 170] were found on DFU. Various kinds of honey products were applied as interventions in the published studies. Different kinds of honey have various active ingredients and concentrations, so they have been excluded from the present analysis. Only one published case series study [103] was found on the use of MH in leg ulcerations with DFU and two RCTs on DFU [104, 105] (Table 4). Gethin and Cowman [103] reported eight cases on the use of MH in leg ulcerations. Only one case involved DFU, while the others involved leg ulcerations with different etiologies. They revealed that the mean initial wound size was 5.62 cm^2^, decreasing to 2.25 cm^2^ at the end of the four-week study. No inclusion and exclusion criteria were mentioned for the study and subject to a high risk of detection bias although the outcome assessor was blinded.

Al Saeed [104] performed an RCT using MH impregnated dressing against tulle on DFU. The result showed that the honey dressing was superior in terms of the proportion of healing, rate of amputation, and time to eradicate the infection. Unfortunately, the methodology of the study was flawed. No report was made on how the randomization, concealment, and double blinding were performed. The criteria for inclusion and exclusion in the study were not stated clearly. The antibiotics that were used and any adverse events were not reported. According to the Cochrane Collaboration's “risk of bias” criteria [171], there was an unclear risk of bias in selection, performance, and detection. Kamaratos et al. [105] performed another RCT using MH tulle against saline soaked gauze. The result was that the intervention group healed significantly more quickly than the control group. An unclear risk of detection bias was found in that the blinding of the outcome assessors was not clearly reported. A high risk of selection bias relating to the randomization sequence was predictable, as the participants were assigned to groups I and II in an alternate manner. The inclusion and exclusion criteria were not clearly reported. Although it was found in both RCTs that MH was more effective than tulle dressing, we were not confident enough to come to a solid conclusion because of the high risk of bias in the design of the research. Therefore, a high-quality study with a vigorously designed RCT for DFU is needed to enrich the body of knowledge in this area.

## 5. Comparison of Nanocrystalline Silver and Manuka Honey Dressing

According to the review in the preceding sections, both nAg and MH have clear antibacterial and anti-inflammatory effects in vitro. In this section, we review the studies conducted in the past 10 years comparing the antibacterial effect, cytotoxicity (Table 5), and clinical effectiveness of silver and honey.

### 5.1. Antibacterial Effect

Nasir et al. [106] conducted an in vitro study to compare the antibacterial effect of hydrofiber Ag (aquacel Ag) and hydrofiber soaked with manuka honey. They found that that hydrofiber Ag had a greater zone of inhibition (ZOI) than MH for Gram-negative bacteria, but no statistical test was performed for the comparison. Guthrie et al. [107] obtained a similar result. They carried out an animal study on a mice traumatic model contaminated with* S. aureus* and revealed that the nAg group had statistically significantly lower bacterial counts than the MH group. However, Bradshaw [108] conducted an in vitro study and obtained a contradictory result. Bradshaw compared different iodine, honey, and silver wound dressings and found no significant overall difference in ZOI among the three groups. Nevertheless, a significant difference in ZOI was observed between different types of Ag dressings against each bacterium. There could be several reasons for the inconsistent findings. First, the nature of the dressing materials, as a carrier medium to hold the active ingredient of Ag or honey, might have affected the antibacterial activity. Second, the origins of the honey used in these studies differed. Third, the chemical nature of silver and its concentrations in different commercial brands varied.

In addition, Lund-Nielsen et al. [109] conducted a single blinded RCT to study the qualitative bacteriology in malignant wounds using honey-coated and silver-coated dressings. No significant differences were found between the groups. However, this result may not be generalizable to other types of wounds since malignant wounds are characterized by continuous tissue deterioration with a large volume of necrotic tissue and slough. This may lead to a high bioburden.

### 5.2. Cytotoxicity

Surprisingly, the in vitro findings comparing the cytotoxicity of silver and honey are inconsistent, since silver has well-known cytotoxic effect, as shown in previous in vitro studies. Du Toit and Page [110] conducted an in vitro comparison of both types of dressing with regard to the cell morphological effects of keratinocytes and fibroblasts. Compared with the honey (30% medical grade honey gel) group and the control (untreated group), the Acticoat (nAg dressing) group had poor keratinocyte and fibroblast cell proliferation. Cell survival, migration, and shape were negatively affected. Tshukudu et al. [111] performed another similar in vitro study on cell viability. A different honey-based (medical grade multifloral honey from Bulgaria) dressing and different silver-based (Ag and nAg) dressings were tested. They found that all the dressing extracts showed variable effects on cell viability and that the exposed cells showed a similar morphology. Acticoat (nAg with alginate) was the most toxic to cells, with less than 30% viability. Interestingly, the group treated with Atrauman silver (Ag tulle contains triglycerides) demonstrated an increase in the number of viable cells as compared with the control group, but we could not establish any solid conclusion on whether the triglycerides contributed to the viability of the cells. In general, nAg still had a marked cytotoxic effect on cells in comparison with the tested honey.

To conclude, the inconsistent findings that were reported on the antibacterial and cytotoxic effects of silver compared with honey dressings might have depended on the types of dressings that were tested. Therefore, the findings from one dressing might not be generalizable to other dressings using similar ingredients.

### 5.3. The Clinical Evidence from Comparisons between MH and nAg Dressings

In vivo comparisons between honey and silver have been very limited. We reviewed the literature from the past 10 years comparing silver and honey. To our knowledge, three RCTs [172–174] and one retrospective study [175] were published on their effect on burns and malignant wounds. Only one comparative study was published on the use of nAg and MH dressings on malignant wounds. The other studies investigated the effect of different types of honey in comparison with silver sulphadiazine on burn wounds; these studies are therefore excluded from analysis in this review. No comparative study was published on nAg and MH in DFU. Lund-Nielsen et al. [174] compared the application of MH and nAg on malignant wounds and found no differences between MH and nAg in wound healing, cleanliness, mal-odor control, and wound pain. The design of this study was good, with clearly reported inclusion and exclusion criteria, randomization procedure, and allocation concealment. Baseline demographic data between the groups were compared prior to the analysis. Unfortunately, this study still contained some methodological flaws. There was no estimation of the power of the required sample size, no blinding of outcome assessors, and a per-protocol analysis was used instead of an intention to treat analysis. The result was a high risk of detection and attrition bias.

## 6. Conclusion

To conclude, from the findings of the in vitro and animal studies, both MH and nAg have clear antibacterial and anti-inflammatory effects. They seem to have the capacity to potentially influence the pathogenetic role of a number of mechanisms that contribute to impaired healing and chronicity of DFU. In addition, the in vitro and animal studies produced inconsistent findings on the relative potency of the antibacterial and cytotoxic effects of silver and MH. This may be due to the different types of dressings that were used, with different concentrations of active ingredients. Importantly, there is limited evidence on the clinical effectiveness of MH, nAg, and nAg versus MH in DFU. Only some limited case studies series and loosely designed RCTs in a related area were found. This literature review points to a clear future direction for research in a related area. Based on the best available in vitro evidence, we can justify our practice of using silver or honey in terms of the molecular science. However, there is no strong evidence to show that there is an absolute clinical benefit to using nAg or MH ingredients as topical dressings to treat DFU. For now, we cannot conclude whether nAg or MH is more suitable for treating DFU. Therefore, there is a need for a vigorously designed clinical study with human participants to investigate the solo effect of MH and nAg and to compare their clinical effectiveness.

## Figures and Tables

**Table 1:** The molecular alternations of DFU.

Number	Author	Nature of study	Cellular abnormalities
[30]	Loots et al., 1999	In vitro	Fibroblasts decrease proliferative capacity and abnormal morphology
[31]	Galkowska et al., 2005	In vivo	Leukocytes decrease accumulation
[32]	Waltenberger et al., 2000	In vitro	Monocytes in diabetic patient are less reactive to VEGF
[33]	Usui et al., 2008	In vivo	Impaired migration and differentiation of keratinocytes
[34]	Albiero et al., 2011	Animal study	Reduced in the recruitment, survival, and proliferation of endothelial progenitors at the site of the injury
[35]	Kanter et al., 2012	Animal study	Decreased in the polarization and activation of macrophages

Number	Author	Nature of study	Poor ECM formation

[29]	Blakytny and Jude, 2006	Review	AGEs cause the upregulation of MMPs and cytokines that degrades ECM through the production of ROS
[40]	Sibbald and Woo, 2008	Review	The overexpression of MMPs and elastase breaks down the components of ECM and inhibits growth factors

Number	Author	Nature of study	High levels of MMPs

[41]	Lobmann et al., 2002	In vivo	MMP-1 and MMP-9 increased 65-fold and 14-fold, respectively, in diabetic ulcer biopsies
[42]	Muller et al., 2008	In vivo	MMP-8 and MMP-9 remained stable in the poor healer group but decreased in the good healer group

Number	Author	Nature of study	High proinflammatory cytokines

[27]	Lobmann et al., 2005	Review	The upregulation of TNF-*α* and IL-1 stimulated the synthesis of MMP-1 and inhibited the synthesis of collagen
[39]	McLennan et al., 2008	Review	Hyperglycaemia activates the pathways of mitogen-activated protein kinase to stimulate cytokine production and promote inflammation
[44]	Trengove et al., 2000	In vivo	IL-1, IL-6, and TNF-*α* are upregulated in chronic nonhealing ulcers
[45]	Chan et al., 2012	In vitro	Neutralization of TNF improves the angiogenesis

Number	Author	Nature of study	High oxidative stress

[47]	van den Berg et al., 2008	In vitro	Free radicals (superoxide anion and hydroxyl radicals) are formed by the oxidative degradation of glycated proteins, which subsequently form AGEs
[49]	Soneja et al., 2005	Review	The production of peroxynitrite anion and peroxynitrous acid can lead to biological damage

**Table 2:** Modes of action of nAg.

Number	Author	Nature of study	Mechanism of antibacterial action
[54]	Kon and Rai, 2013	Review	Increases the surface area contacting the wound surface
[55]	Morones et al., 2005	In vitro	Have a direct interaction on the cell surface and within the bacteria with a diameter of around 1–10 nm
[56]	Pal et al., 2007	In vitro	The bactericidal action of nAg with a truncated triangular shape exceeds that of nAg with a spherical or rod shape
[57]	Matsumura et al., 2003	In vitro	Interacts with the thiol group of respiratory enzymes
[58]	Gordon et al., 2010	Animal study	Inactivates the key enzyme by blinding the thiol group, forming free radicals, and subsequently damaging DNA
[59]	Pandian et al., 2010	In vitro	Interacts with and condenses phosphorus-containing DNA and cytoplasm (apoptosis) and inhibits cell replication
[60]	Dibrov et al., 2002	In vitro	Binds to the modified phospholipid bilayer and induces a massive leakage of protons
[61]	Sondi and Salopek-Sondi, 2004	In vitro	Attaches the negatively charged cell membrane by forming “pits,” making the membrane porous and resulting in leakage of intracellular content
[62]	Kim et al., 2007	In vitro	Bacteria release cellular content after the permeability of the cell membrane increases, leading to cell death
[63]	Mirzajani et al., 2011	In vitro	Destroyed the bonds of glycan strands composed of N-acetylglucosamine and N-acetylmuramic acid in the cell membrane of Gram +ve bacteria and causing “pits” to form
[64]	McQuillan et al., 2012	In vitro	Interacts with the outer and inner membrane of Gram −ve bacteria, and then membrane dissolves; Ag^+^ releases into the cell and affects a transcriptional response
[65]	Mijakovic et al., 2006	In vitro	Phosphorylation of the protein substrate in bacteria can influence bacterial sign transduction and cell cycle progression
[66]	Shrivastava et al., 2007		

Number	Author	Nature of study	Mechanism of anti-inflammatory action

[67]	Wright et al., 2002	Animal study	Reduces the activity of MMPs and stimulates the apoptosis of PMNs, leading to a decrease in the release of cytotoxic compounds such as proteases and oxygen radicals
[68]	Bhol et al., 2004	Animal study	Effectively decreases allergic contact dermatitis on a guinea pig model, similar to topical steroids
[69]	Bhol and Schechter, 2005	Animal study	Suppresses the activities of TNF-*α* and IL-12 and induces the apoptosis of inflammatory cells
[70]	Wong et al., 2009	Animal study	Downregulates the production of TNF-*α* without having a significant toxic effect on a peritoneal adhesion model
[71]	Nadworny et al., 2010	Animal study	Decreases TNF-*α* and IL-8 and increases IL-4, EGF, KGF, and KGF-2
[72]	Nadworny et al., 2010	Animal study	Downregulates TNF-*α* and IL-8 and upregulates IL-4, IL-10, EGF, KGF, and KGF-2
[73]	Bisson et al., 2013	Animal study	Demonstrates a significant inflammatory effect, equivalent to that which results from using topical steroid cream
[74]	Shin et al., 2007	In vivo	TNF-*α* and interferon-*γ* are significantly inhibited at low concentrations of nAg
[75]	Mani et al., 2015	In vivo	TNF-*α*, IL-1*β*, and IL-6 are inhibited at concentrations ranging from 10 to 20 *μ*g/mL

**Table 3:** Modes of action of manuka honey.

Number	Authors	Nature of studies	Major findings on antibacterial action

			*Physical property *
[76]	M. D. Mandal and S. Mandal, 2011	Review	Osmotic effect can draw water from bacteria and dehydrate them
[77]	Molan, 2001	Review	Acidity (pH 3.2–5.5) can inhibit the growth of most microorganisms

			*Active ingredient *
[78]	Adams et al., 2008	In vitro	High concentrations of MGO ranged from 38 to 828 mg/kg as compared with non-MH
[79]	Mavric et al., 2008	In vitro	MGO ranging from 38 to 761 mg/kg can inhibit *E. coli* and *S. aureus* at 1.1 mM
[80]	Atrott and Henle, 2009	In vitro	MGO ranged from 189 to 835 mg/kg and was directly responsible for the antibacterial property
[81]	Kwakman et al., 2011	In vitro	Glycoside of methyl syringate called “Leptosin” correlated positively with antibacterial activity
[82]	Kato et al., 2012	In vitro	Other than MGO, cationic and noncationic compounds contributed to antibacterial activity

			*Mechanism of action *
[83] [84]	Henriques et al., 2010 Jenkins et al., 2011	In vitro	Honey-treated cells fail to proceed cell division and separation (i) *S. aureus*(ii) MRSA
[85]	Roberts et al., 2012	In vitro	Extensive cell lysis on *P. aeruginosa* at MIC 12% (w/v) after 60 minutes of exposure to MH
[86]	Packer et al., 2012	In vitro	Ribosomal function on *S. aureus* interfered including protein synthesis, the metabolic process, and transcription
[87]	Maddocks et al., 2012	In vitro	Inhibition of the binding of *Streptococcus pyogenes* to fibronectin and the development of biofilm
[88]	Kronda et al., 2013	In vitro	Limit *P. aeruginosa* to capture iron and impede its growth

			*Bactericidal activity on different microorganisms *
[89]	Cooper et al., 1999	In vitro	*Streptococcus pyogenes *
[90]	Cooper et al., 2002	In vitro	*Pseudomonas* species
[91]	Hammond and Donkor, 2013	In vitro	*Clostridium difficile *
[81]	Kwakman et al., 2011	In vitro	MRSA, *Bacillus subtilis*, *E. coli*, *P. aeruginosa *
[87]	Maddocks et al., 2012	In vitro	*Streptococcus pyogenes *
[92]	Cooper et al., 2002	In vitro	Vancomycin-resistant *Enterococci *
[93]	French et al., 2005	In vitro	Antibiotic-resistant strains of coagulase-negative *Staphylococci *

Number	Authors	Nature of studies	Major findings on anti-inflammatory action

			*Active ingredient *
[94]	Chan et al., 2013	In vitro	Pinobanksin, pinocembrin, luteolin, and chrysin are the major flavonoids found in MH; low levels of quercetin and galangin were also detected
[95]	Raso et al., 2001	In vitro	Quercetin and galangin inhibit the expression of COX-2 and iNOs in a concentration-dependent manner
[96]	Cho et al., 2004	In vitro	Chrysin suppresses the activity of proinflammatory enzymes
[97]	Tonks et al., 2007	In vitro	5.8-kDa component isolated from MH stimulates the proinflammatory cytokines TNF-*α* and IL-1*β* and the anti-inflammatory cytokines IL-6 via toll-like receptor (TLR) 4
[98]	Tomblin et al., 2014	In vitro	Phenolic content is directly correlated to the anti-inflammatory activity of MH through a TLR1/TLR2 signalling pathway

Number	Authors	Nature of studies	Major findings on anti-inflammatory action

			*Mechanism of action *
[99]	Henriques et al., 2006	In vitro	The formation of free radicals such as hydroxyl radicals are inhibited and contribute to resolving chronic inflammation
[100]	Tonks et al., 2003	In vitro	Proinflammatory cytokines TNF-*α* and IL-1*β* as well as anti-inflammatory cytokine IL-6 from monocytes are stimulated
[47]	van den Berg et al., 2008	In vitro	ROS production and scavenge superoxide anions are inhibited
[101]	Bean, 2012	In vitro	The proinflammatory cytokine TNF-*α* and the anti-inflammatory cytokines IL-10 and IL-1 and the growth factors PDGF and TGF-*β* are upregulated ROS production by phagocytosis is downregulated
[102]	Leong et al., 2012	In vitro Animal study	The production of superoxides by neutrophil decreased Leukocyte infiltration and odema in the mice model decreased

**Table 4:** The clinical evidence on manuka honey topical dressings on DFU.

Number	Author	Nature of study	Number of subjects	Intervention	Funding	Major findings	Comments
[103]	Gethin and Cowman, 2005	Case series	8	Manuka honey	Not stated	A mean initial wound size of 5.62 cm for all wounds decreased to 2.25 cm at the end of the four-week treatment period.	There was a high risk of detection bias because the outcome assessors were not blinded. There was unclear risk of selection bias because no inclusion and exclusion criteria were mentioned. Only one out of eight ulcers was DFU.

[104]	Al Saeed, 2013	RCT	59	Manuka honey impregnated dressing versus tulle	Self-funded	The percentage of ulcers healed in the honey group (61.3%) was significantly higher than in the control group (11.5%). There were significant fewer toe amputations in the honey group (9.7%) compared with the control group (34.6%).	There was an unclear risk of selection and performance bias because randomization, concealment, and double blinding were not reported. Inclusion and exclusion criteria were not clearly stated. The use of any antibiotics and any adverse events were not reported.

[105]	Kamaratos et al., 2014	RCT	63	Manuka honey tulle versus saline soaked gauze	Self-funded	The two groups did not differ significantly in the percentage of ulcers healed at the 16-week follow-up session. The mean healing time in the honey group of 31 ± 4 days was significantly shorter than the 43 ± 3 days for the control group.	There was an unclear risk of detection bias because the blinding of the outcome assessors was not clearly reported. There was a high risk of selection bias because no true randomization was performed. Inclusion and exclusion criteria were not clearly reported. Adverse effects were not reported.

**Table 5:** Comparison of the antibacterial effect and cytotoxicity of honey and silver.

Number	Authors	Funding	Nature of studies	Major findings
				*Antibacterial effect *
[106]	Nasir et al., 2010	University-funded	In vitro	Aquacel Ag (hydrofiber Ag) had a greater zone of inhibition than MH-soaked aquacel in Gram-negative bacteria
[107]	Guthrie et al., 2014	Self-funded	Animal study	Acticoat (nAg dressing) can reduce the bacterial burden more effectively than MH in a heavily contaminated mice model
[108]	Bradshaw, 2011	University-funded	In vitro	There is no significant difference in antibacterial activity between honey and silver dressings, but a significant difference in the strength of activity among different brands of silver dressings
[109]	Lund-Nielsen et al., 2011	Self-funded	In vivo	There is no significant difference in the qualitative bacteriology of malignant wounds between honey-coated and silver-coated dressings

				*Cytotoxicity *
[110]	Du Toit and Page, 2009	Self-funded	In vitro	Marked cytotoxicity with high nonviability staining and cell-scoring was observed in the nAg group (Acticoat) compared with the honey group (L-Mesitran: medical grade natural honey from Netherlands) and the control group
[111]	Tshukudu et al., 2010	Company-sponsored	In vitro	There was no significant difference between the best-performing silver and honey-based dressing extracts with regard to cell viability
